# Acknowledged Signatures of Matrix Metalloproteinases in Takayasu's Arteritis

**DOI:** 10.1155/2014/827105

**Published:** 2014-09-03

**Authors:** Gang Wu, Nitin Mahajan, Veena Dhawan

**Affiliations:** ^1^Department of Orthopedics, Wuhan General Hospital of Guangzhou Command, 627 Wuluo Road, Wuhan, Hubei 430070, China; ^2^Department of Experimental Medicine and Biotechnology, Postgraduate Institute of Medical Education and Research (PGIMER), Chandigarh 160036, India; ^3^Division of Molecular Oncology, Department of Internal Medicine, Washington University in St. Louis, St. Louis, MO 63110, USA

## Abstract

Takayasu's arteritis (TA) was reported as an eye disease in the year 1905 and later was confirmed as a vasculitis. Since then, the etiology of the disease remains unknown; however, characteristic clinical features suggest multiple causative factors. Recent progress in vascular biology and other disciplines enlightens the pathophysiology of TA and demonstrated induction of various nonspecific inflammatory symptoms and destruction of the arterial wall, which leads to aneurysms and rupture of the affected arteries. Matrix metalloproteinases (MMPs) as an enzyme family have well-established roles in several vascular pathologies including intima formation, atherosclerosiss and aneurysms. MMPs have been proposed to be one of the molecules with a potential of having dual role in the course of TA, first as an active participant in pathophysiology and secondly as a diagnostic biomarker for TA disease. The desire to improve our understanding of the importance of MMPs and their endogenous inhibitors (TIMPs) in TA disease and for the development of therapeutic agents has inspired basic and clinical scientists for over a decade. In the present paper, we summarized the scientific rationale which highlights the signatures of matrix metalloproteinases and their endogenous inhibitors in pathophysiology as well as their being a potential candidate as biomarker for Takayasu's arteritis.

## 1. Introduction

Takayasu's arteritis is a chronic vasculitis that affects large vessels and its main branches [1–3]. Mikito Takayasu, a Japanese ophthalmologist reported a case of a young female whose eyes exhibited “coronary anastomosis,” arteriovenous anastomosis around the papilla in 1905, which is the first description of this disease in the literature [4, 5]. Several cases were reported in Japan after this, including a report in 1939 by Yasuzo Shinmi who used the term “Takayasu's arteritis” for the first time [6]. It was later in 1948 when Schimizo and Sano recognized the clinical triad of absent radial pulses, hypertensive carotid sinus, and ocular fundal changes [7]. Clinically varied ischemic symptoms due to stenotic lesions or thrombus formation, including blindness, cataract, and/or retinal haemorrhage, pulselessness, aortic regurgitation, and/or congestive heart failure due to dilatation of the ascending aorta have been reported worldwide. Aneurysms and/or dissecting aneurysm or rupture of the involved arteries due to the destruction of the media of the arterial wall has been commonly reported in the acute condition of the disease [1, 8–12]. Though, a number of studies, experimental and clinical, have been conducted worldwide, the pathophysiology of the disease still remains enigmatic. Factors like infectious agents, autoimmunity, and genetic factors are considered to play a major role in the physiopathology of this disease. Clinical manifestations of TA are highly variable and depend on the stage of disease and distribution of vascular lesions [1, 3]. Significant geographical variations are also noted in the frequency of major clinical features in subjects with TA [7, 13, 14].

Recent epidemiological data are lacking; however, previous studies demonstrated a major prevalence of TA in Asian and Central and South American countries like Japan, India, China, Korea, Thailand, Peru, Israel, Brazil, and Mexico [1, 15–18]. Studies from USA and Sweden estimated incidence rates to be 2.6 and 1.2 cases per million populations per year [13]. TA has been reported to occur predominantly in the females as compared to males [1, 15]. The female and male ratio shows significant variation among different ethnic groups being 8–24 : 1 among Japanese patients and 6 : 1 in Korean [19], 2.1 : 1 in Indian [16], 1.2 : 1 in Israeli, 2.15 : 1 in Thai patients [20] and 8.4 : 1 among the Turkish patients [21] and 6 : 1 in Brazilian [22] and 6.9 : 1 in Mexican patients [23].

Histopathologically TA is characterized as a “panarteritis,” involving all the layers of the arterial wall [1, 2, 24]. In TA, the earliest change appears to be a granulomatous inflammation in the adventitia and outer layers of the affected arteries, followed by gradual progression to a panarteritis with inflammatory mononuclear cell infiltration. The normal architecture of the vessel is disrupted, resulting from histologically characterized degeneration of the elastic lamella in the media of affected elastic arteries. The process eventually produces intimal thickening and scarring and aneurysms form in the weakened arterial walls, particularly when the aorta itself is involved. In this process, proteases (majorly MMPs) secreted from infiltrated cells as well as resident vascular cells come into the play and virtue of their inevitable property to degrade extracellular matrix (ECM) leads to vascular remodeling [2, 24, 25]. Matrix metalloproteinases (MMPs) and their endogenous inhibitors (TIMPs) have been studied to explore their importance in pathophysiology as well as their being a candidate biomarker for Takayasu's arteritis (TA). In the present paper we summarized the scientific rationale which highlights the signatures of MMP-TIMP axis in Takayasu's arteritis.

## 2. Matrix Metalloproteinases

Matrix metalloproteinases are a family of more than 20 structurally related, zinc-containing enzymes that degrade the extracellular matrix (ECM) and connective tissue proteins. Remodeling of ECM components by MMPs affects proliferation, migration, and apoptosis of vascular cells [26, 27]. Under normal physiological conditions these MMPs are under tight control of their endogenous inhibitors, that is, tissue inhibitors of matrix metalloproteinases (TIMPs). An imbalance between MMPs and TIMPs could thus cause increase in the activity of MMPs and, therefore, lead to pathological changes in the vessel wall structure associated with vascular disease [25–28].

**Figure 1:**
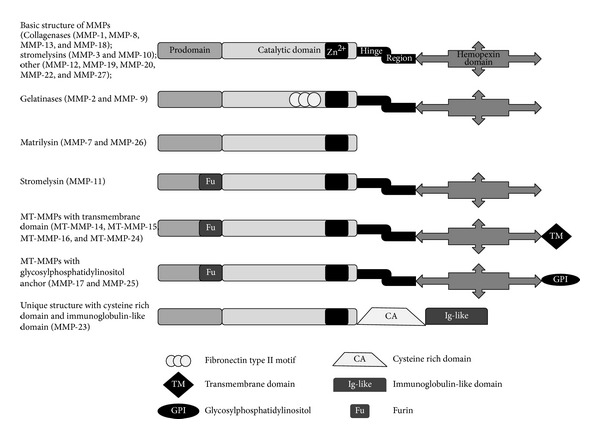
Structure of matrix metalloproteinases (MMPs). MMPs contain four domains, that is, prodomain, catalytic domain, hinge region, and hemopexin domain; however, each MMP has a slight difference in its subunit organization. Catalytic domain has Zn^2+^ binding site. Matrilysins lack hinge region and hemopexin domain. Gelatinases (MMP-2 and MMP-9) contain three repeats of a fibronectin-like motif that bind gelatin. MMPs contain a specific recognition motif for intracellular furin-like serine proteinases (Fu) that allows intracellular activation by these proteinases. Membrane-type MMPs contain a transmembrane domain or a glycosylphosphatidylinositol anchor. MMP-23 has a unique cysteine-rich region and an immunoglobulin-like domain in the place of hemopexin domain.

MMPs typically consist of four domains, that is, propeptide domain (~80 AA long), catalytic domain (~170 AA long), hinge region (of variable length), and hemopexin domain (~200 AA long) (Figure 1). Proteinases assigned to the MMP family have three molecular signatures. (i) Sequence homology with collagenase-1 (MMP-1). (ii) Cysteine switch motif PRCGXPD in the prodomain that maintains MMPs in their pro-MMP zymogen form. The conserved cysteine chelates the active zinc site. MMP-23 has a unique cysteine-rich region and an immunoglobulin-like domain in place of hemopexin domain. (iii) Zinc-binding motif bound by 3 histidine molecules with the conserved sequence HEXGHXXGXXH located in the catalytic domain [27–30]. In normal physiological vascular remodelling, the activities of MMPs are tightly regulated at the transcription level, activation of their proform or zymogens, their interaction with specific ECM components, and inhibition by endogenous inhibitors. Activation of proforms of MMPs involves detaching of the hemopexin domain and requires the action of other MMPs or other classes of proteinases [29, 31].

Currently, 26 members of the MMP family have been identified in vertebrates out of which 23 of them have been found in humans [27–29, 31]. MMPs are divided into the following six groups (Table 1). (i) Collagenases, a group of MMPs, include MMP-1, MMP-8, MMP-13, and MMP-18. (ii) Gelatinases include gelatinase-A (MMP-2) and gelatinase-B (MMP-9), mainly digest denatured collagens (gelatins). (iii) Stromelysins include MMP-3 and MMP-10 and MMP-11. (iv) Matrilysins include matrilysin-1 (MMP-7) and matrilysin-2 (MMP-26, endometase). This group of MMPs lacks the hemopexin domain. (v) Membrane-type MMPs (MT-MMPs) include the type-I transmembrane proteins MT1-, MT2-, MT3-, and MT4-MMP (MMP-14, MMP-15, MMP-16, and MMP-24) and the glycosylphosphatidylinositol- (GPI-) anchored proteins MT5- and MT6-MMP (MMP-17 and MMP-25). MT1-MMP can digest type-I, -II, and -III collagen and other components of ECM. These can also activate pro-MMP to MMP. (vi) Other MMPs include MMP-12, MMP-19, MMP-20, MMP-22, MMP-23, and MMP-28 [27–29, 31].

Tissue inhibitors of matrix metalloproteinases (TIMPs) (21–29 KDa) are specific endogenous inhibitors that bind to MMPs in a 1 : 1 stoichiometry and reversibly inhibit the proteolyitic activity of MMPs. Four TIMPs (TIMP1–4) have been identified so far, and their expression is regulated during development and tissue remodeling. Structurally TIMPs are 40% identical [32] and have an N-terminal domain (125 AA) and C-terminal domain (65 AA), each containing 3 conserved disulfide bonds. The N-terminal domain folds as a separate unit and is capable of inhibiting MMPs [30, 33–35]. Disulfide bonds at conserved cysteine residues maintain the six loop structures. The inhibitory function of TIMPs occurs through the interaction between Zn^2+^ of the MMPs with the N-terminal cysteine residues of the TIMPs [32]. TIMP1, TIMP2, and TIMP4 are known to be localised extracellularly in soluble form, while TIMP3 is reported to be tightly attached to extracellular matrix [32, 36]. However, recent study demonstrated that TIMP3 could also be present in the extracellular space in soluble form [37]. Despite the overlap among TIMPs in their inhibitory functions, each TIMP possesses unique property and inhibits the activity of MMPs with different sensitivity and specificity.

## 3. MMP-TIMP Axis in Pathophysiology of Takayasu's Arteritis

Matrix remodeling is a complex process and is controlled by an intricate network of cells and matrix interactions. Adventitial inflammation is the predominant feature in many forms of arteritis where inflammatory cells cluster in the adventitial space surrounding the afflicted artery [38, 39]. There is growing evidence that MMPs are involved in all stages of the atherosclerotic disease, from the initial lesion to plaque rupture. Increased levels of cytokines within that microenvironment might induce production of MMPs from various resident and inflammatory cells, which results in the destruction of elastic fibers in the arterial media [40, 41]. Despite the evidence, the role of adventitia in vascular lesion formation and in the pathogenesis of Takayasu's arteritis has been controversial [24, 42, 43]. In an important* in vitro* study, Mahajan et al. demonstrated that a direct contact between activated T-lymphocytes (isolated from TA subjects) and HT1080 fibroblast leads to enhanced expression of MMPs. Subcellular fractionation studies with T cells isolated from TA subjects pointed out the presence of some unidentified antigenic moiety in membrane fraction which is responsible for the enhanced expression of MMPs by HT1080 cells [44]. Therefore, interaction of different cells in the adventitial setting generates a microenvironment which channelizes cells to invade the media and intima through adventitia due to augmented MMP expression. Inhibiting the direct interaction of these cells can be a potential target for developing novel biological strategies for modulating the ECM turnover.

Finding enhanced transcriptional expression of MMPs in TA subjects as compared to normal healthy controls, Mahajan et al. emphasized the fact that extracellular matrix remodeling is activated in TA disease, thus making these subjects highly vulnerable to atherosclerosis and development of its related manifestations and aneurysms [44]. In corroboration, increased serum levels of MMPs and gelatinolytic activities of MMPs have been demonstrated in TA subjects [14, 17, 44–47]. Various cytokines and chemokines, like interleukin-6 (IL-6), regulated on activation normal T cell expressed and secreted (RANTES), and monocytic chemotactic protein-1 (MCP-1), are reported to increase in patients with TA and therefore could possibly accelerate the production of MMPs by inflammatory [16, 47, 48].

The potential role of reactive oxygen (ROS) and nitrogen (RNS) species to influence ECM remodeling though activation of MMPs is well versed in different vascular disease settings [29]. A positive correlation of increased oxidative stress with levels of MMPs in TA has been demonstrated previously [14]. A negative association of soluble receptor for advanced glycation end products (sRAGE; an antiatherogenic molecule) with MMPs in TA has also been documented [17] and, therefore, provides another evidence for the importance of MMPs in pathophysiology of TA.

## 4. MMP-TIMP Axis as Biomarker in Takayasu's Arteritis

In the natural course of TA, patients experience cycles of active and remission phases that reflect the different inflammatory states of the arterial lesions [2]. Monitoring of disease is very important to check the activity as well as to choose the appropriate therapeutic strategy. Though, prognosis and vascular complications of TA are improving day by day, still development of a single or multiple biomarker approach is desirable. Matrix metalloproteinase has been studied widely in various diseases as a potential disease biomarker. As stated earlier, TA is associated with a significantly increased risk and a higher prevalence of atherosclerosis. Cardiovascular complications including cardiac failure, pulmonary hypertension, cerebrovascular incident, myocardial infarction (MI), and ruptured aortic aneurysm are the major causes of morbidity and mortality [1, 2, 9, 18]. Increased levels of MMPs have been reported in various vascular and vasculitic pathophysiologies like giant cell arteritis [49], Kawasaki disease [50], atherosclerosis and related complications [31, 51], Henoch Schonlein purpura (HSP) subjects [52], and so forth.

Matsuyama et al. have demonstrated that MMP-2 levels were higher in patients with TA than in controls, but no correlation was found between serum MMP-2 and disease activity score [46]. Based on their results these authors concluded that MMP-2 is a helpful biomarker in the diagnosis of TA but not for determining the phase of the disease. On the contrary, recently Ishihara et al. did not find any significant difference in the levels of MMP-2 when compared between TA subjects and controls in Japanese population [53].

MMP-3 levels were also reported in TA subjects in different studies, though contrary. Levels of MMP-3 were found to be significantly high in TA subjects compared to controls [46, 47], whereas, in other studies authors did not observe any significant difference in the levels in TA subjects and controls [17, 53]. In an isolated report by Mahajan et al. MMP-1 levels were found to be insignificant between controls and TA subjects [17]. Treating TA subjects with prednisolone resulted in an increase in serum levels of MMP-3 [53, 54], whereas Matsuyama et al. reported no impact on the MMP-3 levels [46]. The mechanisms by which steroids affect MMP-3 serum levels remain unknown; therefore, determining the MMP-3 level to assess TA disease activity requires more caution.

Serum levels of MMP-9 were found to be significantly higher in TA patients as compared to controls [17]. Matsuyama et al. reported significantly augmented levels of MMP-9 in TA subjects in active phase as compared to healthy controls. Similar findings were also reported in other studies [46]. Patients with TA who were in remission had significantly higher MMP-9 levels than normal controls. Thus, evaluation of MMP-9 may suggest prior existence of TA even in inactive patients, although the mechanism of this elevation is a matter of further investigation. Also, MMP-9 was not affected by the prednisolone dose [53]. On the contrary, Mahajan et al. did not find any significant difference in the levels of MMP-9 in controls versus patients as well as active versus in remission TA subjects [17].

In a study reported from Japan, TIMP1 levels were found to be significantly high in control subjects as compared to those TA subjects who were in active disease [46]. On the contrary, a significantly high level of TIMP1 in subjects with TA as compared to the levels of healthy control subjects was observed in an Indian study cohort [17]. Further, TIMP1 levels remained statistically higher in TA subjects in remission when the data was compared with controls. Higher levels of this endogenous inhibitor of MMPs in TA subjects in this study were explained as a compensatory or adaptive phenomenon. Augmented levels of TIMP1 in TA subjects could be to combat or to compensate and counteract the deleterious effects caused by increased expression of MMPs to some extent in TA subjects. Increased levels of TIMP1 have been observed in patients with coronary artery disease [51, 55] and in subjects with myocardial infarction [56].

Currently a number of clinical imaging techniques have been developed and those are able to correlate with MMP expression in vascular diseases. In an important study where outcomes of MRI were analyzed with MMP-9 levels, Sun et al. have observed that the serum levels of MMP-9 were significantly associated with the severity of lumen stenosis and wall thickness in TA subjects [47]. Also, these authors emphasized the fact that MMP-9 has more strong and significant association with MRI outcome compared to other markers like ESR and hsCRP, which had a moderate association [47], suggesting it might be useful to predict the imaging outcomes of the patients with TA using soluble markers. Moreover, imaging techniques of MMPs have also been advanced in the last decade [57, 58]. Such imaging techniques, if refined in near future, can be used to track the expression of MMPs* in vivo*, which will be useful to monitor both disease progression and therapeutic efficacy.

## 5. Futuristic Approach and Concluding Remarks

The management of TA is challenging and includes medical therapy with steroids, immunosuppressive agents, and revascularization procedure. Despite the large number of studies with the aim to develop agents that target MMP-TIMP axis, none have yet been reached to the market for clinical use. The major caveat is the incomplete understanding of the various pathways regulating MMP-TIMP axis in vascular diseases. Moreover, most of the studies assessing this particular axis in the pathogenesis of vascular disease are extrapolated from either animal models or majorly* in vitro* studies, which may be inaccurate when reached at the door of a clinic. Minocycline, an inhibitor of MMPs, in combination with steroids, was reported to induce clinical remission in TA subjects [59]. Extracellular matrix metalloproteinase inducer (EMMPRIN), a transmembrane glycoprotein, is capable of inducing MMPs through direct cell-cell interaction [60]; therefore, strategies that inhibit EMMPRIN expression can be explored as a promising therapeutic target in future. Of note, MMP inhibitors developed so far lack specificity and are associated with number of side effects.

As stated earlier a number of factors (like genetic predisposition, pathogens, and autoimmunity) have been considered to play role in pathophysiology of disease and, therefore, to assess the disease activity and plan treatment strategy become difficult. In addition, patients who are in remission may develop new arterial lesions along follow-up. Therefore, it has always been a challenge in TA research to assess the disease activity. Also, most of the systemic manifestations are not specific to vasculitis and moreover vascular feature progresses very slowly. Undoubtedly, imaging modalities allow earlier and accurate diagnosis; however, serum biomarkers have their own advantages over imaging, that is, cost effectiveness, less cumbersome, and less time to analyze the reports. Therefore, studies focusing on the association of clinical imaging techniques with MMP expression in vascular disease will be the need of this hour. Such studies will allow not only risk stratification, but also analyzing the effects of any MMP-TIMP axis targeted therapy.

The best method to diagnose TA would be one that involves a multiple biomarker approach rather than a single biomarker approach. Though some biomarkers show high sensitivity or specificity, it should be noted that small-scale verification in retrospectively selected samples from one institute might not represent a general test with sample prospectively collected from multiple institutions. Therefore the combination of multiple biomarkers that best distinguish TA samples from controls should be utilized in the development of TA detection technology for clinical applications.

We would like to emphasize that TA has been reported all over the world, with wide variation in its prevalence in different geographical areas; therefore, a small sample size along with a single-center-based is the inherited limitations for the studies which report such a rare vascular disorder. Most of the studies reported so far are hypothesis generating and therefore lack a definitive and firm conclusion. Therefore, meta-analysis and the multi-institutional studies will be of importance. Based on the review of literature (as summarized in Table 1), MMPs and TIMPs cannot be applied for the routine diagnostic of TA yet and the clinical utility of these molecules remains to be established. However, there is evidence that MMPs contribute to the TA pathology and may be targets for future therapeutic strategies using selective inhibitors. Therefore, future studies concerning the significance of selected MMPs and their inhibitors, as potential biomarkers for TA, need to be continued.

## Figures and Tables

**Table 1:** Summary of the various MMPs and TIMPs and alterations in their levels in subjects with Takayasu's arteritis.

Subgroup	MMPs/TIMPs	Observations	Reference
Collagenases	MMP-1 (collagenase-1)	N.S	[17]
		TA > C, A > R (mRNA)	[44]
	MMP-8 (collagenase-2)	—	—
	MMP-13 (collagenase-3)	—	—
	MMP-18 (collagenase-4)	—	—

Gelatinases	MMP-2 (gelatinase-A)	N.S	[53]
		TA > C, A > R (Activity)	[14]
	MMP-9 (gelatinase-B)	A > C	[46]
		TA > C	[17]
		TA > C; A > R	[47]
		A > R > C	[53]
		TA > C, A > R (mRNA)	[44]
		TA > C, A > R (Activity)	

Stromelysins	MMP-3 (stromelysin 1)	A > C	[46]
		N.S	[17]
		TA > C	[47]
		N.S	[53]
		TA > C, A > R (mRNA)	[44]
	MMP-10 (stromelysin 2)	—	—
	MMP-11 (stromelysin 3)	—	—

Matrilysins	MMP-7 (matrilysin-1)	—	—
	MMP-26 (matrilysin-2)	—	—

Membrane-type MMPs	MMP-14, MMP-15, MMP-16, MMP-17, MMP-23, MMP-24, and MMP-25	—	—

Other MMPs	MMP-12, MMP-19, MMP-20, MMP-22, and MMP-28	—	—

TIMPs	TIMP1	C > A	[46]
		TA > C, R > C	[17]
		C > TA	[47]
		TA > C, A versus R = N.S (mRNA)	[44]
	TIMP2	—	
	TIMP3	—	
	TIMP4	—	

TA: total TA population; A: active; R: remission; C: controls; N.S: not significant.
